# Horizontal jump asymmetries are associated with reduced range of motion and vertical jump performance in female soccer players

**DOI:** 10.1186/s13102-023-00697-1

**Published:** 2023-07-08

**Authors:** Alberto Roso-Moliner, Demetrio Lozano, Hadi Nobari, Chris Bishop, Antonio Carton-Llorente, Elena Mainer-Pardos

**Affiliations:** 1 Health Sciences Faculty, Universidad San Jorge, Autov A23 Km 299, Villanueva de Gállego, 50830 Zaragoza, Spain; 2 Department of Exercise Physiology, Faculty of Educational Sciences and Psychology, University of Mohaghegh Ardabili, Ardabil, Iran; 3 Faculty of Sport Sciences, University of Extremadura, 10003 Cáceres, Spain; 4 Faculty of Science and Technology, London Sport Institute, Middlesex University, London, NW4 1RL UK

**Keywords:** Symmetry, Bilateral asymmetry, Jump, ROM, Injury risk, Football

## Abstract

**Background:**

Performance in jumping and change of direction tests are good proxies to reflect the skill level during soccer-specific actions. Greater inter-leg asymmetries have been identified as a risk factor for developing acute and overuse injuries and jeopardizing soccer performance. The aim of this study was to assess the association between asymmetry in the unilateral vertical and horizontal jump tests, ankle range of motion, linear velocity, and change of direction in a sample of highly trained adult female soccer players.

**Methods:**

Thirty-eight highly trained female soccer players underwent a testing protocol including ankle dorsiflexion, single leg jumps for height (CMJ), distance (HJ), 40 m sprint and 180° change of direction tests.

**Results:**

Within-session reliability was acceptable (CV ≤ 7.9%), and relative reliability showed good to excellent (ICC: 0.83 to 0.99). The one-way ANOVA reported higher inter-limb differences for change of direction deficit (10.9 ± 8.04%) and single leg CMJ (5.70 ± 5.22%). Pearson correlations highlighted significant relationships between horizontal jump asymmetries and ankle dorsiflexion (*r* = -0.41), CMJ (*r* = -0.36 to -0.49) and HJ (*r* = -0.28 to -0.56).

**Conclusions:**

Assessing inter-limb asymmetries through different methods can help scientists understand the specificity of their detrimental effects on soccer performance. Practitioners should be aware of these specificities as well as the magnitude and direction of the asymmetries when aiming to improve specific on-field skills.

## Introduction

The growth of women's soccer is a priority in FIFA's strategy, which has set a goal of doubling the number of participants between 2014 and 2026 [1]. Consequently, this growing interest has also increased research into the female game [2]. Performance in soccer requires a high level of physical fitness and mastery of sport-specific technical and tactical skills. It is an intermittent, high-intensity sport, and players must perform repeated sprints, changes of direction (COD), vertical and horizontal jumps, accelerations and decelerations [3, 4]. Consequently, these actions are often unpredictable and thus, frequently result in an uneven distribution of loading on each limb [5].

With this in mind, there has been a significant increase in studies investigating limb-to-limb asymmetry, which refers to the difference in function or performance of one limb relative to the other [6, 7]. For example, when measuring lower body asymmetries during strength tasks, exercises such as squats [8], isokinetic dynamometry [9, 10], and isometric tasks (e. g. the mid-thigh pulldown or the isometric squat) have been used [11, 12]. Although practical, some tests are expensive and time-consuming, making them difficult to use in field settings. Therefore, other methods of assessing limb-to-limb asymmetry that are less time-consuming and costly may be needed. Jump tests provide a practical and rapid form of physical assessment, often mimicking movement patterns (i.e. triple extension of lower limb joints) observed in sports [13, 14]. Previous research has used bilateral and unilateral tests to measure asymmetries, such as the horizontal jump (HJ) [15, 16] and the countermovement jump (CMJ) [15, 17]. Therefore, with a range of protocols previously used to measure asymmetry and the importance of this measure, practitioners should consider which is most appropriate for their athletes based on analysis of the needs of the sport, the players' training history and previous familiarity with the protocols. As noted, not all tests require much time, skill, or investment of money. In summary, assessing asymmetries of specific muscles or muscle groups is an important measurement that could help clarify the origin of possible asymmetries in specific performance tasks.

On the other hand, existing scientific evidence has identified ankle dorsiflexion restriction as an important risk factor for suffering acute sports-related injuries, such as anterior cruciate ligament rupture [18], as well as for developing overuse disorders such as patellar [19], and Achilles tendinopathies, shin splints [20], and anterior patellofemoral pain syndrome [20]. Consequently, it seems likely that ankle dorsiflexion range of motion (ROM) restrictions should be one of the key factors to control in this sport, as it can contribute to knee valgus momentum during landing tasks and squatting movements [20], which, in turn, increases un-wanted force demands higher up the kinetic chain at the knee [21].

In recent years, more research has examined the association between asymmetry and some physical performance tests [15, 16, 22]. For example, Maloney et al. [23] demonstrated that jump asymmetry during the unilateral jump test was associated with lower COD (*r* = 0.6) performance in healthy adult males. Similarly, Bishop et al. [24] demonstrated that unilateral CMJ asymmetry of 12.5% was associated with decreased linear sprint and jump performance in youth soccer players. In contrast, Lockie et al. [16] found no effect on COD and linear velocity performance associated with a unilateral CMJ asymmetry of 10.4% in male collegiate players.

For its part, Dos'Santos et al. [15] found an asymmetry in the unilateral HJ of 6.3% that showed no association with COD performance in a male university sample. Furthermore, to the authors' knowledge and rather importantly, few studies exist in populations of adult female soccer players [25, 26]. Of note, the cdcstudies mentioned above found jump tests are more sensitive for identifying between-limbs asymmetries (8 to 12%) than COD tests (2 to 3%) and were negative related with sprint times [25, 26]. These contradictory results indicate the need for further research to establish the relationship between these asymmetries and measures of physical performance.

Because of the above, the main objective of the present study was to demonstrate the relationship between asymmetry in the unilateral vertical and horizontal jump tests (CMJ and HJ), ankle range of motion, linear velocity, and COD in a sample of highly trained adult female soccer players. Given the paucity of data assessing the association between asymmetry and physical performance in female populations, it was challenging to elucidate a suitable hypothesis.

## Materials and methods

### Study design

A cross-sectional design was used in this study. Informed consent was provided to all female soccer players following the policy of their soccer club after approval by the Clinical Research Ethics Committee of the Government of Aragon (PI21/011, CEICA, Spain) and respecting the ethical standards of the Declaration of Helsinki.

### Participants

Thirty-eight female soccer players (age: 23.5 ± 3.6 years; height: 163.9 ± 5.4 cm; body mass: 60.1 ± 5.5 kg; body mass index: 22.4 ± 2.3 kg/m^2^) participated in this study. All soccer players belonged to the same women's soccer division (Spanish Second Division) and had performed structured weekly training in terms of volume and methodology (i.e. four sessions of 90 min duration and 1 match per week) for a minimum of 10 months and had at least three years of experience in semi-professional soccer. In addition, the female soccer players were injury-free at the time of the test and during the 3-month intervention.

### Procedures

Test sessions began with a rise, activate, mobilize, and potentiate system warm-up protocol [27]. Each participant was then asked to complete three unilateral CMJ and HJ preparation jumps at progressive intensities (i.e., 60%, 80% and 100% of perceived exertion) in preparation for both tests. During the second testing session, three practice trials of a 40 m sprint and a 505 COD test were completed at the same perceived intensities. These submaximal trials served as a familiarization test in the field. A passive rest period of three minutes was provided before data collection. The tests were conducted the first few days of the following week of the intervention, at the same time (17:00–19:00) and under identical ambient conditions (22 °C and 20% humidity). Participants were instructed to refrain from vigorous activity for at least 48 h before data collection, and all tests were conducted on a soccer field with soccer boots.

#### Weight-bearing dorsiflexion ROM

The LegMotion system (LegMotion, your Motion, Albacete, Spain) was used to assess ankle weight-bearing dorsiflexion mobility (WB-DF), and the test is described elsewhere [28]. Each player performed three trials with each ankle to make the most appropriate measurement, recovering 10 s between each attempt.

#### Countermovement unilateral and bilateral jump

All jumps were performed on the soccer field and in soccer boots. All participants initiated each jump with hands on hips, with a leg countermovement to a depth chosen by the user before accelerating vertically and jumping as high as possible. For unilateral jumps, the leg used had to remain fully extended during the flight phase before landing on the floor. Jump height in centimetres was recorded using the “My Jump 2” smartphone app. Two jumps were performed with the right leg and two with the left leg, resting for 60 s. The highest jump was selected for further data analysis.

#### Standing long jump test

A standard 30-m tape measure (30 m M13; Stanley, New Britain, USA) was used. The subjects started the test standing with their feet placed behind the line and their arms relaxed. They were instructed to jump the maximum possible horizontal distance, executing a controlled landing and maintaining balance on the performing leg or both legs (3 s) until the evaluator recorded the fall position. The length was measured from the jump line to the rearmost heel in the subject's landing. Two unilateral jump attempts were performed with each leg, and two bilateral jump attempts. Each with 60 s of recovery between jumps, the best unilateral and bilateral jump was recorded for later analysis.

#### 40 m sprints

Photocell timing gates (Witty, Microgate, Bolzano, Italy) were placed at 0, 10, 20, 30, and 40 m. Participants started the test upright, putting their feet 30 cm behind the starting line [29]. Two attempts were made on the soccer field, separated by a rest period of 180 s. Times were recorded in hundredths of a second. The best time was used for subsequent analysis.

#### 505 Change-of-Direction speed test and Change-of-Direction deficit

Photocell gates (Witty, Microgate, Bolzano, Italy) were placed at 10 and 15 m. Subjects sprinted 15 m over the soccer field and performed a 180° turn with both the right and left leg, completing two trials for each leg. The time analyzed was from the 10 m photocells, the 180° turn once past the 15 m photocells, and the sprint back through the timing gates over the 10 m mark. A 180-s rest period was provided between attempts. As described by Shepard et al. [30], the best time of the right and left leg was used for further data analysis. The COD deficit (CODD) was calculated from the difference between the times of the first part of the linear sprint (i.e. time recorded in 10 m) and the COD test (i.e. 505 test) [31, 32].

### Statistical analysis

SPSS statistical software (Version 25.0; SPSS Inc, Chicago, IL, USA) was used. The Shapiro–Wilk test was used to determine normality. Within-session reliability of test measures was calculated using the coefficient of variation (CV) (absolute reliability) and a two-way random intraclass correlation coefficient (ICC) (relative reliability) with the complete agreement and 95% confidence intervals. The ICC values were interpreted using Koo et al. guideline’s [33], and a CV < 10% as an acceptable responsibility criterion. It should be mentioned that asymmetries may favour either side depending on whether limb scores are higher [34]. Therefore, the consistency of the direction of asymmetry between tests was measured using the Kappa coefficient and they were interpreted as poor (≤ 0), slight (0.01–0.20), fair (0.21–0.40), moderate (0.41–0.60), substantial (0.61–0.80), almost perfect (0.81–0.99) and perfect [35]. The following equation calculated inter-limb asymmetries as the percentage difference between the two limbs [36].

$$100/\mathrm{Max\;value\;}(\mathrm{right\;and\;left}) *\mathrm{ Min\;value\;}(\mathrm{right\;and\;left}) * - 1 + 100$$

A one-way repeated measures ANOVA determined the systematic bias between mean asymmetry values. Pearson's correlations were performed between inter-limb asymmetry scores and performance tests. Statistical significance was inferred from *p* < 0.05.

## Results

Table 1 contains information on within-session reliability. Flexibility, jump and COD tests revealed good to excellent reliability (ICC: 0.83 to 0.99) and acceptable variability (CV ≤ 7.9%). After comparing the mean asymmetry of the groups, no significant results were observed.

**Table 1:** Mean scores, mean asymmetry (%) and reliability data for each test

Test^a^	Mean ± SD	Asymmetry (%)	CV (%)	ICC (95% CI)
Unilateral WB-DF (cm)				
WB-DF_R_	42.8 ± 4.71	3.65 ± 3.54	2.7	0.95 (0.90–0.97)
WB-DF_L_	42.02 ± 5.11		1.3	0.99 (0.98–0.99)
Unilateral CMJ (cm)				
CMJ_R_	13.6 ± 1.68	5.70 ± 5.22	1.7	0.98 (0.96–0.99)
CMJ_L_	13.6 ± 1.39		2.4	0.95 (0.90–0.97)
Unilateral HJ (cm)				
HJ_R_	146.4 ± 12.1	2.12 ± 2.10	2.1	0.93 (0.86–0.96)
HJ_L_	147.2 ± 11.3		1.9	0.94 (0.88–0.97)
Sprint				
10 m	1.93 ± 0.19	-	4	0.83 (0.70–0.91)
20 m	3.36 ± 0.21		1.9	0.88 (0.78–0.94)
30 m	4.72 ± 0.29		2.3	0.87 (0.76–0.93)
40 m	6.13 ± 0.37		2.1	0.86 (0.74–0.92)
COD				
COD_R_	2.62 ± 0.16	2.99 ± 2.22	2.6	0.87 (0.76–0.93)
COD_L_	2.62 ± 0.18		2.1	0.92 (0.86–0.96)
CODD_R_	0.70 ± 0.24		7.9	0.91 (0.85–0.95)
CODD_L_	0.69 ± 0.25	10.9 ± 8.04	7.3	0.93 (0.88–0.96)

^a^*ROM* Range of motion, *CMJ* Countermovement jump, *HJ* Horizontal jump, *COD* Change of direction, *R* Right, *L* Left, *SD* standard deviation, *CV* coefficient of variation, *ICC* intraclass correlation coefficient, *WB-DF* Weight-bearing dorsiflexion test, *CODD* Change of direction deficit

Table 2 shows the Pearson’s correlations between inter-limb asymmetry scores and performance tests. No significant relationships were found between CMJ and COD asymmetry and the mobility, jumping, sprinting and COD tests. On the other hand, ankle dorsiflexion ROM asymmetry was significantly correlated with CMJ right (*r* = -0.36) and 10 m sprint time (*r* = 0.37). In addition, HJ asymmetry was correlated with WB-DF (*r* = -0.41; for both legs), CMJ (*r* = -0.36; *r* = -0.49; right and left leg, respectively) and HJ right (*r* = -0.56). Finally, CODD asymmetry was statistically associated with HJ left (*r* = -0.46).

**Table 2:** Correlations between range of motions, jump and change of direction tests and asymmetry percentages

Test^a^	WB-DF Asymmetry	CMJ Asymmetry	HJ Asymmetry	COD Asymmetry	CODD Asymmetry
WB-DF_R_	0.12	-0.08	-0.41**	0.05	0.08
WB-DF_L_	-0.12	-0.07	-0.41**	0.16	0.14
CMJ_R_	-0.36*	-0.16	-0.36*	0.04	0.08
CMJ_L_	0.18	-0.15	-0.49**	0.18	0.16
HJ_R_	-0.18	-0.10	-0.56**	-0.01	0.07
HJ_L_	0.14	0.07	-0.28	-0.27	-0.46**
10 m	0.37*	-0.08	0.27	-0.01	0.15
20 m	-0.29	-0.04	0.19	0.12	0.23
30 m	-0.24	-0.06	0.16	0.14	0.27
40 m	-0.21	-0.03	0.17	0.18	0.29
COD_R_	-0.15	-0.14	0.20	0.08	-0.01
COD_L_	-0.06	-0.10	0.22	0.06	-0.08
CODD_R_	0.15	-0.06	-0.09	0.18	-0.12
CODD_L_	0.20	-0.11	-0.05	0.14	-0.17

^a^*ROM* Range of motion, *CMJ* Countermovement jump, *HJ* Horizontal jump, *R* Right, *L* Left, *SLROM* Single leg range of motion, *COD* Change of direction, *WB-DF* Weight-bearing dorsiflexion test, *CODD* Change of direction deficit

**Fig. 1:**
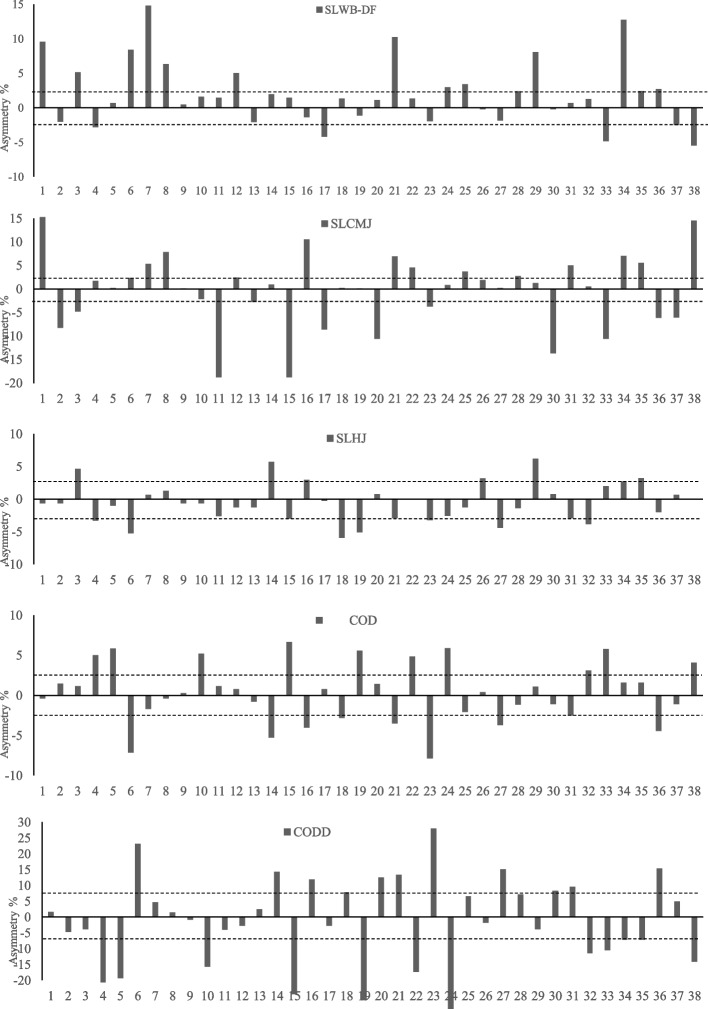
Individual asymmetry data for all performance tests (above 0 indicates right leg dominance and below 0 indicates left leg dominance) SLCMJ: Single leg countermovement jump; SLHJ: Single leg horizontal jump; 180° COD: 5 + 5 sprint test with a 180°; SLWB-DF: Single leg weight-bearing dorsiflexion test; CODD: Change of direction deficit

Table 3 shows the levels of agreement for the asymmetry scores (Kappa coefficient). The results showed reasonable levels of agreement between the WB-DF test and the single leg CMJ (0.29) and slight levels between single leg CMJ and HJ (0.11), CODD and single leg HJ (0.10), single leg CMJ (0.02) and WB-DF (0.02). The rest of the test’s comparison shows poor levels (-0.87 to -0.13). Individual inter-limb discrepancies are illustrated in Fig. 1 for WB-DF, HJ, CMJ, COD and CODD due to the variable nature in both the magnitude and direction of asymmetry.

**Table 3:** Kappa coefficients and descriptive levels of concordance of asymmetries between the jumping speed and COD tests

Test Comparison^a^	Kappa Coefficient	Descriptor
SLWB-DF -SLCMJ	0.29	Fair
SLCMJ-SLHJ	0.11	Slight
SLHJ-COD	-0.31	Fair
SLWB-DF -COD	-0.13	Poor
SLWB-DF -SLHJ	-0.21	Poor
SLCMJ-COD	-0.13	Poor
SLHJ-CODD	0.10	Slight
SLCMJ-CODD	0.02	Slight
SLWB-DF-CODD	0.02	Slight
COD-CODD	-0.87	Poor

^a^*SLROM* Single leg range of motion, *SLCMJ* Single leg countermovement jump, *SLHJ* Single leg horizontal jump, *SLWB-DF* Single leg weight-bearing dorsiflexion test, *COD* Change of direction, *CODD* Change of direction deficit

## Discussion

The present study examined the relationship between asymmetry in the horizontal and vertical jump, ankle dorsiflexion, linear velocity and COD in highly female soccer players. The results showed significant correlations between ankle dorsiflexion ROM and vertical jump performance with HJ asymmetry. The results suggest that more enormous HJ imbalances were associated with lower range of motion in ankle dorsiflexion and reduced vertical jump height. In addition, the asymmetries between ankle dorsiflexion (WB-DF), vertical jump (CMJ), horizontal jump (HJ), change of direction (COD, and CODD) rarely favored the same side, signifying the task-specific nature of asymmetry.

Table 1 shows that the metrics of inter-limb asymmetry differ depending on the test used. Therefore, female players do not all react similarly to the same asymmetry test. Literature has shown that asymmetries of > 10% may decrease jump height [37] and increase COD speed times [38]. indicating that the reduction of these differences may be favourable. The first aspect to note is that higher percentages of asymmetries were identified for the CMJ than for the HJ (5.70% vs. 2.12%). Previous studies in young female soccer players [26] or recreational team sports players [16], reinforce our results, finding 12.54% and 10.4% asymmetries in the vertical jump versus 6.79% and 3.3% in the HJ, respectively. Like the authors cited above, it is thought that the vertical jump could be more sensitive than the HJ when identifying asymmetries. One explanation for this is that the movement patterns related to HJ are practised from an earlier age than those of vertical jumping [26]. In our study, the sample is composed of highly trained female soccer players, and this level of training could explain why the percentages of asymmetry in both jump tests are lower than in the other two studies cited. This is confirmed when we observe a ratio of asymmetries in the studies with adult female elite soccer players of 8.65% [39] or 7.9% in elite male players [40]. This could also explain the mean values found for the percentage of asymmetry of the ROM, which are lower (3.65%) than those obtained in studies such as that of Madruga-Parera et al. (5.88%) [41]. The CODD follows this same trend, as our data show somewhat lower asymmetries (10.9% ± 8.04) than those found in pre-season female soccer players (24%) [42] and young players [43]. Finally, the data obtained for COD asymmetry (2.99%) are very similar to those found in other studies of adult female soccer players (2.39%) [39] or young players [43]. Further research is needed to confirm these theories regardless of the results obtained.

In Table 1, we can also observe good to excellent reliability (ICC: 0.83 to 0.99) and acceptable variability (CV ≤ 7.9%), indicating that the data can be interpreted with confidence for further analysis [44]. Again, the level of the players in our sample and their physical preparation could explain this acceptable level of variability, and the reliability obtained. Haugen et al. [45] reported a CV of 3.26% for bilateral CMJ using a force platform and a CV from 1.82% to 3.30% in 40 m linear sprints measured each 10 m. In other studies such as that of Bishop et al. [17], the CV for unilateral CMJ is 2.82% and 3.51% (right and left leg, respectively), and for HJ from 3.94% to 4.18% (right and left leg, respectively). In our case, for these tests, we obtained a CV ranging from 1.7% to 2.4% for the unilateral CMJ (right and left leg, respectively), from 2.1% to 1.9% for the unilateral SH (right and left leg, respectively), and from 1.9% to 4% for the 40 m linear sprint (measured every 10 m). In the Madruga-Parera et al. [41] study, a CV of 1.70% and 1.94% was obtained for the Ankle Dorsiflexion test and 2.88% and 2.05% for the COD test (right and left leg, respectively), results very similar to those obtained in the present study. Finally, regarding the CODD, we received a CV of 7.9% for the right leg and 7.3% for the left leg. Previous studies show results that do not differ much from those found in the present investigation (female cricketers: 8.6% for both legs; female netball players: 4.9% left leg and 6% right leg; female soccer players: 12.7% left leg and 6.6% right leg) [42]. We believe that our sample's training and fitness level again explains the similar CV for both legs. Exell et al. [46] suggest that asymmetries can only be natural if the differences between the limbs are more significant than the variability during the test protocols. In the present study, all CV values fulfilled this criterion. Therefore, all observed differences can be considered accurate. In addition, previous studies recommend performing several measurements, calculating test variability and searching for lower CV values (acceptable < 10% or CV < 5% in unilateral jump tests), in the process of continuous monitoring of professional players [39, 47], which the authors of the present article also recommend.

Table 2 shows the Pearson correlations between the limb asymmetry scores and the measurements performed (unilateral WB-DF, unilateral CMJ, unilateral HJ, Linear speed 40 m measured every 10 m, COD and CODD). One of the main findings in the present study is the significant relationship between SH asymmetry and decreased performance in the CMJ right (*r* = -0.36), CMJ left (*r* = -0.49), HJ right (*r* = -0.56), WB-DF right (*r* = -0.41) and WB-DF left (*r* = -0.41). Recent publications report that unilateral jumping performance factors (including asymmetries) are direction-specific and affect the performance of the weaker leg [26]. This assumption would partially explain the results obtained in the present work for HJ, but it does not hold for the vertical jump since the asymmetries in CMJ were not correlated with decreased unilateral jumping and cutting performance.

To our knowledge, few research have associated asymmetries between horizontal and vertical jumps. Significant correlations have been found between the percentage of asymmetry in the vertical and the HJ (*r* =—0.489; *p* < 0.05) [48]. Regarding the significant relationship between HJ asymmetry and decreased ankle dorsiflexion mobility (*r* = -0.41, both for right and left leg), we have also not found any previous research that has associated such asymmetries. However, Barrera-Domínguez et al. [49], indicate that a more significant asymmetry in dynamic balance as measured by the Y-Balance Test is associated with a lower ankle ROM and a decrease in performance in both horizontal and vertical jumping. We think all these results may be because this type of jump (e.g., HJ) is not trained as much within the specific physical preparation of female soccer players or they are not so decisive in competitive actions.

A significant relationship was also found between ankle ROM asymmetry and performance in the CMJ right (*r* = -0.36) or linear velocity measured in the first 10 m (*r* = 0.37). The study by Godinho et al. [50], shows a positive correlation between jumping ability (e.g., CMJ) and higher ankle ROM. This study also establishes a relationship between ankle dorsiflexion and dynamic balance in athletes. This aspect supports the systematic review carried out by Mesfar et al. [51], which indicates that there may be a specific association between the asymmetries of this dynamic balance and jumping performance and, on the other hand, the range of movement of the ankle and the COD tests in that involving a 180° turn. Other studies have also confirmed this, which have found significant relationships between ankle ROM and improved dynamic balance or lower body motor skills [52, 53]. About linear velocity, some studies establish a positive relationship between adequate ankle ROM and improved linear speed or centre of mass acceleration in adult sprinters [54, 55]. This provides evidence that adequate ankle dorsiflexion may be related to improved performance in the unilateral CMJ, the COD and the first few metres of the linear sprint. However, we have found little literature associated with the study of ankle ROM and performance, especially in women's soccer, and more research is needed to confirm these findings.

Finally, CODD asymmetry was significantly related to decreased HJL performance (*r* = -0.46). The literature that has analysed CODD asymmetry associated with different types of jumps is very scarce, and, in most cases, bilateral hops have been used. For example, Bishop et al. [56] analysed the % asymmetry in the CODD and compared it with different bilateral jumps (squat jump, CMJ, and drop squat). No significant relationships were obtained, but it was observed that the greater the asymmetry in the CODD, the lower the performance in the different jumps. The most similar results to those observed in the present study have been found in a recent study [57], which has negatively associated the CODD with the jump height of the right leg (*r* = -0.59) and the concentric impulse of the same leg (*r* = -0.60). Our results would reinforce the idea of those obtained in this research and could support the concept that greater competence in the unilateral jump means better performance in the CODs. In addition, greater concentric strength could also improve this performance, as the ability to apply this type of force is decisive during the turning phase of the 505 tests.

No significant associations were found between CMJ, COD asymmetries, and the fitness mentioned above tests. These data are consistent with those presented by Lockie et al. [16] and Dos'Santos et al. [58], in which no associations were observed between CMJ asymmetry and jumping tests or time reduction in the COD (i.e., 505) in male collegiate athletes. Nor did Azahara et al. [5], find significant associations between CMJ asymmetry and total time during the agility (V-Cut) test in young male and female athletes playing team sports. The same study found significant relationships between CMJ asymmetry and 30 m linear velocity, which disagrees with our results. However, the magnitude of this relationship was small (*r* = 0.26; *p* < 0.05). Regarding adult female soccer players, Bishop et al. [39] found no correlation between vertical jump asymmetry (CMJ), 30 m linear velocity (measured at 0 m, 10 m and 30 m) and COD (505) performance. This is similar to the results shown in the study by Loturco et al. [59], in which they found no association between vertical jump asymmetries and linear velocity (30 m sprint measured every 5, 10, 20, and 30 m) and COD (zig-zag test) in adult elite female soccer players. The lack of association of vertical jump and COD asymmetries with soccer-specific performance tests in the present study may be related to the players' high level of general and specific training on this type of action [60]. In addition, a large part of the competitive actions in soccer is related to these vertical jumps or COD, which could minimise the effects of asymmetries on performance [6, 7].

Kappa coefficients were used to assess the direction of the asymmetry. Through this test, it can be observed whether the asymmetry favours one limb or the other. The results in Table 3 show fair levels of agreement between the WB-DF test and the same leg CMJ (0.29) and slight levels of agreement between the unilateral CMJ and the same-leg HJ (0.11). In addition, the one-leg CODD also showed a slight level of agreement with the same leg HJ (0.10), CMJ (0.02) and WB-DF (0.02). The rest of the test comparison shows poor levels (-0.87 to -0.13), indicating that the relationship favours the opposite leg. The individual differences between limbs are illustrated in Fig. 1, representing the magnitude and direction of the asymmetries. Bishop [61] also suggested that the guide of the asymmetry and its importance could help to understand which limb performed better, as an absolute positive value of the asymmetry would not allow identification of the asymmetry. All of tThe above suggests that differences between limbs do not favour the same leg in different tests. Based on these results and previous research, the detection of asymmetries in adult female soccer players should be performed individually and using a single test for their evaluation is not recommended [6, 62].

A possible limitation of the present study is that the sample was not divided by playing position due to the small number of players. Other studies, with larger samples, divide the players by playing positions to analyse their differences [6, 62, 63]. Secondly, the Y-balance test asymmetries have been shown to be negatively correlated with CMJ height [64]. Therefore, this dynamic balance test could be included in future studies. Given that the push-off process for a COD and HJ are similar, this type of vertical jump could be added in future research because it may be more sensitive to COD performance. In addition, data cannot be extrapolated to other populations such as men’s soccer, amateur level or other team sports. Despite these considerations, the present work provides relevant data on lower limb asymmetries in certain performance variables in female soccer players.

## Conclusions

The present study analysed lower limb asymmetries regarding mobility, jumping and cutting in highly trained female soccer players. In summary, the asymmetries found were greater in the vertical jump and 180° COD tests. However, the asymmetries in the HJ showed the greatest agreement with the reduction in mobility, jump and COD performance. Knowing the relationships between asymmetries and performance can help coaches customize training plans for specific on-field improvements. However, the direction of asymmetry appears highly variable among female soccer players, therefore, practitioners should consider individualized analysis and including different asymmetry tests when planning an intervention program.

## Data Availability

The data presented in this study are available on reasonable request from the corresponding author. The data are not publicly available due to privacy reasons.

## Abbreviations

COD: Change of direction
HJ: Horizontal jump
CMJ: Countermovement jump
CV: Coefficient of variation
ICC: Intraclass coefficient correlation
WB-DF: Ankle weight bearing dorsiflexion
ROM: Range of motion
SLROM: Single leg range of motion
SLCMJ: Single leg countermovement jump
SLHJ: Single leg horizontal jump
SLWB-DF: Single leg weight-bearing dorsiflexion test
